# Study on Survival and Sustainable Development of Small- and Medium-Sized Tourism and Hospitality Enterprises in Macao Based on Regional Soft Environment and Competitive Advantage

**DOI:** 10.3389/fpsyg.2021.817695

**Published:** 2022-01-26

**Authors:** Dongshu Cheng, Kunyuan Liu, Zichao Qian, Ziyang Chen, Honglin Mao

**Affiliations:** ^1^International Tourism Management, Faculty of International Tourism and Management, City University of Macau, Macao, Macao SAR, China; ^2^Financial Management, Faculty of Finance, City University of Macau, Macao, Macao SAR, China; ^3^Business Administration, Faculty of Business, City University of Macau, Macao, Macao SAR, China

**Keywords:** soft environment, competitive advantage, quantitative research, sustainable development, SMEs, business method

## Abstract

The small- and medium-sized tourism and hospitality enterprises are the main forms of enterprises in Macao. This study put forward a new framework of survival and sustainable development from the perspective of competitive advantage and regional soft environment, which significantly holds theoretical and practical research value. The study obtained cross-sectional data of 317 small- and medium-sized tourism and hospitality enterprises in Macao through a large-scale questionnaire survey. This article used exploratory factor analysis and confirmatory factor analysis to test the reliability and validity of each factor system and its measurement scale. The variance analysis is used to judge the differences between the elements. The regression analysis is used to verify the hypothesis of a causal relationship between factors. The structural equation analysis is used to fit the model and make necessary amendments. The results reveal that the government service environment has a significant positive impact on the ability of an entrepreneur. In addition, the regional soft environment significantly impacts the competitive advantage of small- and medium-sized tourism and hospitality enterprises in Macao. Contrary, the impact of laws of Macao on the acquisition of financial resources and the promotion of entrepreneur ability and innovation ability is found insignificant. In conclusion, the results showed that market of Macao lacks venture investors where enterprises cannot obtain funds through angel financing or risk financing.

## Introduction

A sustainable economic development in Macao has attracted much attention from the political, business, and academic circles. The small- and medium-sized enterprises (SMEs) in Macao are an important part of its economic development. Their survival and sustainable development are closely related to the overall economic growth of Macao. It affects the economic structure and economic development speed of Macao. Supporting and promoting the development of SMEs has become a key issue in economic development.

According to the Macao Yearbook of Statistics (2019), tourism and hospitality industry in Macao contributes to the main gross domestic product (GDP) income of Macau and solves the employment problem in Macao. According to the “China Mainland to Macao Online Tourism Market Development Report” released by the China International Trade Promotion Council Taiwan, Hong Kong, and Macao Service Center in 2019, since the 20th anniversary of return of Macao, the number of tourists in Macao has increased by 400%, the mainland supports 70% of total tourists in Macao. As of 2018, the annual GDP of Macao was 440.3 billion Patacas and the tourism revenue of Macao was 373.57 billion Patacas, a year-on-year increase of 13.9%, accounting for 84.84% of GDP of Macao. In the rapid development of the entire tourism and hospitality industry in Macao, although foreign-funded multinational companies and large Chinese-funded companies account for half, the remaining market share is occupied by many SMEs in Macao. At present, the contribution rate of small- and medium-sized tourism and hospitality enterprises in Macao to GDP is about 20–25%. According to the Macao Doctoral Think Tank forecast, by 2030, the economic benefits contributed by the small- and medium-sized tourism and hospitality industry in Macao are expected to rise from the current 20% to about 50%.

In summary, the current economy in Macao depends on tourism and hospitality. There are a large number of SMEs. Research on the survival and sustainable development of small- and medium-sized tourism and hospitality companies in Macao is a matter of concern to the government and society and a question of maintaining a long-term stable and prosperous development in Macao. Therefore, this article takes “Research on the survival and sustainable development of small- and medium-sized tourism and hospitality enterprises in Macao.”

This article reviews the relevant theories of SMEs. It analyzes the process, characteristics, and performance of small- and medium-sized tourism and commercial enterprises in Macao. In particular, this research outlines the systematic approach of Macao to the tourism and hospitality industry, thereby examining the development needs of SMEs. Paying attention to the prosperity of SME, this article presents empirical research on the sustainable development of tourism and hospitality sector in Macau. Sustainability gaining increasing relevance, the survival of SMEs largely depends on different factors. Accordingly, this study provides a comprehensive analysis of the regional environment conditions of the country (i.e., social, cultural, local, governmental, economic) where competitive advantage is necessary to develop a sustainable environment.

However, the recent literature shows that Macao holds an attractive tourism market and is recently facing numerous economic challenges. Over the years, sustainable development in the tourism and hospitality industry has affected the entire economy and environment of Macau. The growth of small to medium-sized businesses has tremendously decreased. As a result, sustainability in tourism and hospitality industry in Macau has become a serious topic for the entrepreneurs who aim to use this study for economic improvement. Therefore, this study holds immense theoretical significance and practical value. As a result, the most economies in the world are paying attention to the potential of tourism and economic competitiveness. An increased focus on SMEs provides innovative information to the professionals and entrepreneurs, guiding them for future development. This study is a widespread achievement enabling the practitioners to ensure the effective infrastructure development of foundation in Macau by radically addressing the emerging economic issues.

This study aims to start with the whole and take the overall development of small- and medium-sized tourism and hospitality industry in Macao as research objective. This article makes a comprehensive and in-depth study on the factors and mechanisms of the survival and sustainable development of small- and medium-sized tourism and hospitality enterprises. It could describe objectively and comprehensively the development process and current situation of the small- and medium-sized tourism and hospitality industry in Macao after the researcher summarizes and analyzes the common limiting factors encountered in the development of small- and medium-sized tourism and hospitality industry in Macao. The researcher explores the deep-seated problems that generate these restrictive factors.

### Development of Small- and Medium-Sized Enterprises in Macao

Although SMEs account for 99% of the total enterprises in Macao, they create more than 60% of employment opportunities, but at present, the impact on the economy in Macao is not great; the development of the industry is difficult and lacks vitality. Portugal has governed Macao for one hundred years, but never paid attention to and supported SMEs; in addition, Macao has always been led by gaming tourism and entertainment and has become the main source of government revenue; this industry accounts for nearly 80% of local taxes, which is nearly 50% of GDP. Therefore, gaming, tourism, and entertainment have a large market share, while SMEs have a very small market share. The Macao SAR government has launched some financing guarantee plans to support the development of SMEs. However, because SMEs have been on the verge of survival for a long time, there are not enough conditions to enjoy the subsidy scheme of the government. Therefore, the operation of SMEs has not been effectively improved.

This study investigates the articles of Macao scholars on SMEs in Macao and the articles reported by Macao newspapers. The difficulties of SMEs in Macao are summarized as follows:

• Market financing ability is poor.
• There is a lack of professional management and supervision.
• The business scope is relatively concentrated.
• Government tax is low, but the ability of profit is common.
• Operating capacity is general.
• There is a lack of support from the local supporting manufacturing industry.

### Research Content

There is an obvious interactive relationship between the soft regional environment and the competitive advantages of small- and medium-sized tourism and hospitality enterprises in Macao. This article focuses on the mechanism of the soft regional environment on the competitive advantage of SMEs in tourism and hospitality in Macao. In the research process, based on extensive reading and systematic summary of competitive enterprise advantage, soft regional environment, and related research literature, this article builds an element function model of the regional soft environment on the competitive advantage of SME. The related research content mainly includes the following three aspects.

(1) The theoretical and empirical analysis of the competitive advantage factor of SMEs is in the stage of theoretical analysis. This article mainly analyzes the three academic theories of the competitive advantage of enterprises: exogenous (industrial structure), endogenous (internal resources, capabilities, and knowledge), and internal and external integration (dynamic capabilities), as well as the respective emphasis on the enterprise competitive advantage factor system and other existing research results of the systematic summary and induction. This article considers the significant industry, scale, and other scenario dependence of competitive advantage of the enterprises. It puts forward the competitive advantage factor system for SMEs in the empirical analysis stage. Fundamentally, this article first designs the corresponding measurement scale and then modifies the scale through interviews with experts, entrepreneurs, and prediction tests. Then, this article takes the small- and medium-sized tourism and hospitality enterprises in Macao as the research sample. Through exploratory factor analysis and confirmatory factor analysis, the reliability and validity of their competitive advantage factor architecture and its measurement scale is verified. One-way analysis of variance (ANOVA) was used to analyze the differences in their competitive advantages.

(2) The theoretical and empirical analysis of regional soft environment elements system is in the stage of theoretical analysis. This article mainly analyzes and generalizes the theory and evaluation system of enterprise ecosystem, enterprise social capital, business system, innovation system, Institute for Management Development (IMD) international competitiveness evaluation theory, and evaluation system, as well as the theoretical and empirical research results of domestic and foreign scholars on the regional economy, science and technology, investment, culture, and other soft environment fields. This article fully considers the situation dependence of the regional soft environment under the background of Macao. From the competitive advantage of small- and medium-sized tourism and hospitality enterprises in Macao, this article puts forward the corresponding system structure of regional soft environment elements.

(3) The theoretical and empirical analysis of the mechanism of the soft regional environment. This part of the research content is the focus of this article. It is based on the above two parts. It is mainly based on previous research results in related fields. This article makes a comprehensive and systematic analysis of the possible significant factors of the soft regional environment on the competitive advantages of small- and medium-sized tourism and hospitality enterprises in Macao. Thus, this article constructs a theoretical model of the role of regional soft environment in Macao on the competitive advantage factors of small- and medium-sized tourism and hospitality enterprises in Macao and puts forward the corresponding research hypotheses. In the stage of empirical analysis, this article first uses regression analysis to preliminarily verify the causal relationship of each factor proposed in the research hypothesis. In this article, structural equation modeling is used to verify the causal relationship between potential exogenous variables and endogenous potential variables. In the structural equation model analysis process, the initial model will be modified step-by-step according to the model fitting results. This is to obtain the final model with good fitting and the test results of each research hypothesis.

## Literature Review

The study main direction is survival and sustainable development of SMEs in tourism and hospitality. This study focuses on reviewing and evaluating the existing competitive advantage theory. Competitive advantage theory makes everyone better off at all levels (i.e., individual, corporation, local, and international). The competitive advantage theory allows businesses to embrace competitive business practices necessary for the development of the firms. The theory rests on the notion that competitive advantage benefits the national economies. It fundamentally leads the SMEs to gain market success. As a significant factor determining the enterprise success, competitive advantage theory formulates a model for the responsibility of firms, ensuring the business survival and success.

### Industry Structure View

The industry structure view provides a systematic analysis tool with the complete structure for studying competitive enterprise advantage (Porter, 1997). Hardwick and Dou (1998) believed that the cost efficiency and operation efficiency of an enterprise determine its competitive advantage. Nachum et al. (2001) believed that the comparative advantage of the country or a region where the enterprise is located still has an important impact on the competitive advantage of an enterprise. These scholars ignored the internal differences of the enterprise. Their research method still does not break through the limitations of neoclassical economics. Therefore, it is difficult to explain why the performance difference among enterprises in the same industry is sometimes even greater than that among enterprises in different industries.

#### Resource-Based View of the Firm

The resource-based view (RBV) theory states that an enterprise is a collection of a series of resources. Its internal resources and accumulation are the key to explaining that an enterprise can obtain excess profits and maintain its competitive advantage. RBV has considerably gained popularity over the years. This theory has gained immense growth and prominence with the competitive advantage model development. RBV provides the firm with a sustainable advantage over its competitors (Barney, 1991).

The theory suggests that a valuable resource (i.e., human resource) of a firm is imitable and rare. Valuable resources enable the organizations to exploit strategic business opportunities, yielding a competitive advantage that is rare and imitable. These unique resources help the firms to survive in the competitive market. SME and competitive advantage are fundamental ideas highlighting the importance of maintaining strategic resources in achieving sustainable development. This strategic factor generates market value by gaining maximum returns. It provides the firm superior ability to compete with the competitors. Utilizing the strategic resources (e.g., RBV), the firms integrate the fundamental element of entrepreneurship in achieving a sustainable competitive advantage.

RBV translates the theoretical phenomenon of the firms into business reality by enhancing the capability of the firms in achieving differential benefits. In particular, RBV of the firm pays too much attention to the assets (tangible or intangible) and capabilities of the firm. However, it ignores the impact of the regional environment on enterprises and does not consider the policy, natural environment, and social and cultural factors.

#### Competence Theory of the Enterprise

Selzniek first proposed the competition theory of the enterprise. According to this theory, enterprise capability is a kind of special capital contained in the enterprise (Loasby, 1995). Enterprise capability is the productivity to determine the combination of resources. Enterprises can use resources effectively and interact with each other to produce new capabilities and resources. The Chinese economist named Jing-lian (2011) believed that a core competence of an enterprise is the ability to organically combine its skills, capital, management, and operation. It results from the two-way effect of internal and external management. It is also a combination of knowledge and skills of the enterprise itself. Therefore, the core competence theory holds that not all the knowledge, resources, and capabilities of an enterprise can form a sustainable competitive advantage.

#### Knowledge-Based View

Knowledge is a special strategic resource. Enterprise is a collection of knowledge. The knowledge possessed by enterprises determines the difference between resource utility and capability. Innovation is just the arrangement and combination of the existing knowledge of an enterprise. Therefore, the competitive advantage of an enterprise is endogenous in its knowledge. The ability and competitive advantage of an enterprise are determined by its knowledge and cognitive learning ability (Grant, 1996). However, there are also some deficiencies in the research of resource, ability, and knowledge-based view of competitive advantage. The knowledge-based view focuses more on the study of the individual level. It is a relatively lack of research on the enterprise level. At the same time, the theory appeared late. Although there are many case studies, they have not yet formed a clear analytical framework and tools.

### Review of Regional Soft Environment Research

The soft regional environment is a relatively complex concept. Research by domestic and foreign scholars on enterprise ecosystem, business system, enterprise social capital, regional innovation system, and other fields related to the external environment of enterprises is closely related to the regional soft environment, and its research ideas have great reference value for the research of regional soft environment.

#### External Environment Theory

Enterprise external environment has always been one of the key research directions in strategic management. The strategic management theory regards the external environment as the economic resources and symbolic resources of enterprises. The exogenous and dynamic capability theories in the theory of enterprise competitive advantage attach great importance to the influence and function of the external environment on enterprise competitive advantage. Kokkali and Koutsouris (2009) believed that the general environment of enterprises mainly includes macroeconomic environment, political and legal environment, social and cultural environment, technological environment, and globalization trend.

#### Enterprise Ecosystem Theory

Singh (1994) believed that the enterprise niche is the characteristics of the enterprise in terms of resources and capabilities. It is the state of the enterprise after the interaction and matching with the environment. Zhang et al. (2020) divided the ecological environment of enterprises into three parts, namely, economic ecology, social ecology, and natural ecology. This prominent approach emphasizes managing the demands of uncertain and changing environments, thus ensuring an organizational sustainability. It seeks to explain the social, economic, and political conditions that affect the overall composition of an organization.

#### Social Capital Theory of Enterprises

Rajan (2002) and Chance (2019) emphasized that good corporate social capital can accelerate the generation and effective transfer of tacit knowledge. The theory of corporate social capital makes us realize that the external environment (especially the regional soft environment), such as the external social network, plays an important role in the internal resources, capabilities, and knowledge of enterprises. Cope et al. (2011) believed that corporate social capital is one of the most important enterprise network resources. The social capital of enterprises can be divided into internal and external. External social capital is the social network that exists outside the enterprise and helps the enterprise absorb all kinds of scarce resources. It includes vertical and horizontal links of enterprises. Its vertical connection refers to the connection between an enterprise and its superior leading organs, local government departments, and subordinate enterprises and departments. Its horizontal connection refers to the connection between enterprises and other enterprises, scientific research institutes, universities, financial institutions, and intermediary organizations.

#### Regional Innovation System

The regional innovation system has gained immense popularity over the past few decades. Regional innovation is a novel way of increasing the growth of SMEs. It increases the competitiveness of the firms by adopting innovative activities and practices. It significantly contributes to the economic development of a nation by strengthening the competitiveness of SME. Accordingly, Crotti and Misrahi (2017) emphasized the important role of the regional innovation system in cultivating enterprise innovation ability. This role is mainly reflected in the innovation environment (especially the innovation culture environment). They believed that the innovation performance of the economy depends not only on the individual enterprise but also on the interaction between enterprises and between enterprises and other institutions. An innovation system is a social system. Innovation is the result of interaction between economic actors in society. As a result, governments are taking initiatives toward developing innovation capabilities, thus supporting the competitiveness of the firms.

### Review of Regional Soft Environment and Competitive Advantage

Barney (1991) emphasized the importance of network resources. He defined network resources as a special enterprise resource used to perceive and implement enterprise strategy. Amit and Schoemaker (1993) thought that the government, the public, and other non-market factors are the same as the competitors, customers, and other market factors. They have a significant impact on the success or failure of enterprises, because they have mastered all kinds of resources that enterprises need for survival and development. Ostroff (1999) believed that the behavior choice of any economic organization is influenced not only by technology, information, and profit in the sense of economics and management but also by various factors in the sense of sociology, such as cultural habits and customs. Therefore, there is an objective interaction between cognition and behavior between enterprises and social factors. Crotti and Misrahi (2017) believed that improving enterprise innovation ability and the acquisition of competitive advantage requires the establishment of close cooperation and learning relationship between enterprises related to various innovation activities.

The research on the effect mechanism of the regional soft environment on the competitive advantage of SMEs mainly focuses on the empirical research of a specific problem, such as regional industrial cluster environment and competitive advantage of small- and medium-sized manufacturing enterprises, financial environment and financing problems of SMEs, and regional development environment problems of SMEs. Scholars pay more attention to the comparative study between different regions.

Guohong and Lijun (2005) and Alfiero and Vallone (2019) established the evaluation model of competitive enterprise advantage, including regional learning, R&D investment, and other factors. Their research results show that the competitive advantage of SMEs largely depends on the absorption, transformation, and innovation ability of knowledge in the region; the technical cooperation and connection between enterprises; and the cooperation and connection between enterprises and universities, scientific research institutes, market intermediary organizations, government, and other factors closely related to the soft environment of regional education and technology (Ajaz et al., 2020; Shah et al., 2021). Gersick (1994) believed that changes in the external environment will make enterprises more actively understand the environment. Guoquan (2001) and Ruosen and Sha (2019) made a detailed analysis of the evolution of the theory of the relationship between organization and environment. They believed that the relationship between enterprise and environment is complex and dynamic. This kind of relationship can be called cooperative competition (Sarfraz et al., 2021). Schroeder et al. (2002) and Castellani et al. (2015) believed that the government should help enterprise clusters carry out breakthrough innovation through normal operation.

## Research Method

### Construction of Competitive Advantage Elements System

The competitive advantage elements system is the relationship between enterprise resources, abilities, and knowledge. Bandura and Bandura (1986), Grant (1996), Lubit (2010), Lorenzo et al. (2018), Larsson and Thulin (2019), Zhang (2019), and other scholars have made corresponding discussions on it. Based on the above definition of the relationship between enterprise resources, capabilities, and knowledge, this article puts forward the corresponding construction ideas of the competitive advantage factor system of SMEs in tourism and hospitality in Macao.

(1) The competitive advantage factor system is composed of resources and capabilities. Knowledge is no longer listed separately but integrated into resources and capabilities.
(2) Resources and capabilities should be clearly defined in the element system.

### Construction of Regional Soft Environment Elements System

Although the regional soft environment of enterprises is not directly studied, the research perspective, methods, and content of IMD provide a good reference for studying the regional soft environment and its element system. This article introduces the evaluation system of IMD and the theoretical and empirical research on the soft environment of regional economic development, regional science and technology development, and regional investment. They have direct reference value for studying regional soft environment and their element system.

### Methodology

**FIGURE 1:**
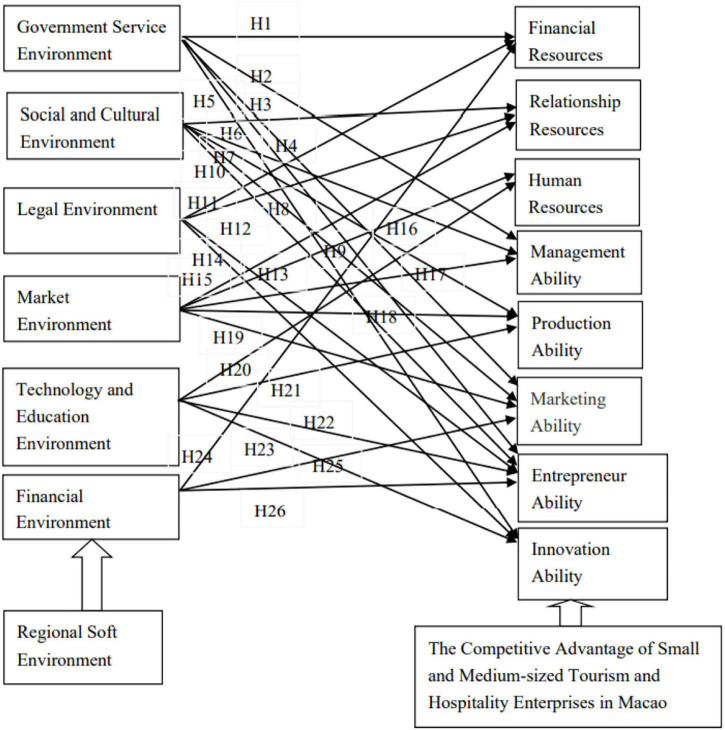
Model of regional soft environment on SMEs in tourism and hospitality in Macao.

This article analyzes the mechanism of the regional soft environment elements on the competitive advantage of SMEs through literature reading and logical reasoning. This study constructs a theoretical model of the effect mechanism of the regional soft environment on the competitive advantage of small- and medium-sized tourism and hospitality enterprises in Macao (Figure 1). This article puts forward the corresponding research hypothesis. Further empirical research needs to verify the theoretical model and research hypothesis.

## Empirical Research

### Research Data

This study considered 520 participants, including shareholders, managers, and practitioners of small- and medium-sized tourism and hospitality enterprises in Macao. The questionnaires were distributed through mobile phones and printed paper. The researcher made the respondents complete the questionnaires either online or off-line and then return them by using the “questionnaire star” website to publish questionnaires and on-the-spot questionnaires. All personal information of respondents was strictly kept confidential. To ensure the reliability and accuracy of data analysis, the questionnaire adopts the methods of monitoring the response time (not less than 100 s) and eliminating the questionnaire with too many same options. Table 1 shows the distribution of the questionnaire, in which 520 questionnaires were sent out and 429 were collected correctly. There are 317 valid questionnaires obtained after screening. The effective rate was 73.89%. A total of 317 valid questionnaires were collected, which exceeded the minimum sample size requirement by three times. At the same time, the maximum likelihood (ML) method is used to estimate the structural model, and the data used are also required to confirm the normal distribution. This article tested all questionnaire items by using the Harman’s single-factor test. The explanation rate of variance of the first component was 19.068%, which is less than 50%. Therefore, the test results showed that the influence of common method biases is not serious in this study.

**TABLE 1:** Common method biases test analysis.

Component	Initial eigenvalues		
	Total	% of Variance	Cumulative%
1	12.585	19.068	19.068
2	5.019	7.604	26.673
3	3.445	5.219	31.892
4	2.926	4.433	36.324
5	2.58	3.909	40.233
6	2.377	3.601	43.835
7	2.287	3.465	47.299
8	2.189	3.317	50.616
9	1.917	2.904	53.521
10	1.612	2.443	55.963
11	1.597	2.419	58.383
12	1.526	2.312	60.695
13	1.443	2.186	62.881
14	1.248	1.891	64.772
			

Table 2 shows the Kaiser-Meyer-Olkin (KMO) value, which is 0.877. It shows that the data are suitable for exploratory factor analysis. It is higher than 0.9. It means that the items in the scale are consistent, the structure of the scale is good, and the reliability is high.

**TABLE 2:** KMO and Bartlett’s sphericity test of competitive advantage.

Kaiser-Meyer-Olkin Measure of Sampling Adequacy	0.877

### Correlation Analysis

Table 3 shows that the correlation coefficient between the innovation ability and the marketing ability of small- and medium-sized tourism and hospitality enterprises in Macao is 0.099. Their correlation is not significant. There is a significant positive correlation between the other factors. The AVE square root value of each factor in the competitive advantage system is greater than the correlation coefficient of the dimension, indicating that the measurement model of the competitive advantage factor has sufficient aggregate validity and discriminant validity.

**TABLE 3:** Correlation analysis of competitive advantage.

Variable	H	F	R	M	P	C	X	E
Human resources	0.741							
Financial resources	0.344**	0.794						
Relationship resources	0.297**	0.319**	0.751					
Management ability	0.302**	0.279**	0.400**	0.736				
Production ability	0.412**	0.236**	0.402**	0.391**	0.817			
Innovation ability	0.354**	0.114*	0.174**	0.172**	0.239**	0.707		
Marketing ability	0.194**	0.360**	0.302**	0.307**	0.312**	0.099	0.683	
Entrepreneurial ability	0.269**	0.317**	0.340**	0.256**	0.354**	0.127*	0.329**	0.717

*p < 0.05; **p < 0.01.

### Confirmatory Factor Analysis

**FIGURE 2:**
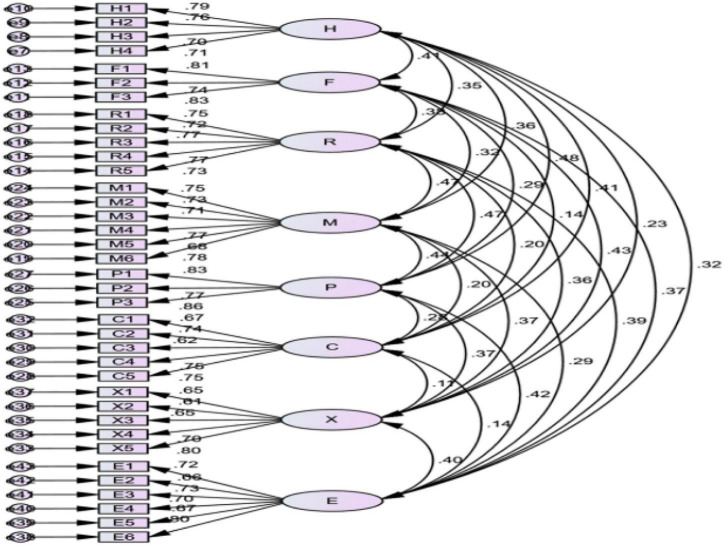
The competitive advantage measurement model.

The main parameters and fitting index are shown in Table 4 and Figure 2. It is shown that the standardized path coefficient of each measurement item under the competitive advantage factor system of SMEs is between 0.11 and 0.48. The standardized path coefficient of each element is between 0.61 and 0.81.

**TABLE 4:** Fitting index of competitive advantage measurement model (*N* = 317).

Fit	χ^2^	df	χ^2^/df	RMSEA	PGFI	IFI	TLI	CFI
Model	771.299	601	1.283	0.03	0.757	0.967	0.963	0.967
Criteria			<5	<0.08	>0.5	>0.9	>0.9	>0.9

The fitting results in Table 4 show that the competitive advantage factor system model of small- and medium-sized tourism and hospitality enterprises in Macao has a good fit. The research model is effective.

### Confirmatory Factor Analysis of Second-Order Model

Although the first-order measurement model of competitive advantage factor system of SMEs is effective, it is more complex. Therefore, the researcher tries to make a simpler model. It is assumed that there is a common second-order factor of the competitive advantage of SMEs, which affects the performance of each first-order factor. That means, 37 parameters reflect the correlation among 8 first-order factors and are replaced by 8 parameters.

Table 5 shows the confirmatory factor analysis results of the second-order factors extracted from the measurement model of the competitive advantage. It shows that the second-order model of competitive advantage factor system of small- and medium-sized tourism and hospitality enterprises in Macao is matched well. Therefore, the second-order model can effectively replace the first-order model.

**TABLE 5:** Fitting index of second order model for competitive advantage (*N* = 317).

Fit	χ^2^	df	χ^2^/df	RMSEA	PGFI	IFI	TLI	CFI
Model	820.797	621	1.322	0.032	0.778	0.961	0.958	0.961
Criteria			<5	<0.08	>0.5	>0.9	>0.9	>0.9

### Empirical Analysis on the Regional Soft Environment of Small- and Medium-Sized Enterprises in Tourism and Hospitality

The KMO measurement and Bartlett’s sphericity test show 29 variables in the measurement scale of the regional soft environment factor system of SMEs in tourism and hospitality in Macao. The KMO value was 0.867, showing that the data are suitable for exploratory factor analysis. Table 6 shows that the correlation coefficient between the average value of each factor of the regional soft environment of small- and medium-sized tourism and hospitality enterprises in Macao is between 0.215 and 0.383. This shows a positive correlation between the elements, and the common variation between the elements is not very high. Therefore, the results of the exploratory analysis can be analyzed in depth.

**TABLE 6:** Correlation analysis of regional soft environment.

Variable	G	S	L	U	T	N
Government service environment	0.730					
Social and cultural environment	0.308**	0.683				
Legal environment	0.352**	0.295**	0.731			
Market environment	0.263**	0.259**	0.264**	0.696		
Technology and education environment	0.237**	0.338**	0.215**	0.350**	0.746	
Financial environment	0.358**	0.297**	0.383**	0.395**	0.324**	0.699

**p < 0.01.

In the research model, there are 6 factors, including government service environment, technology and education environment, market environment, social and cultural environment, legal environment, and financial environment, extracted by exploratory factor analysis represent the source factors of regional soft environment of small- and medium-sized tourism and hospitality enterprises in Macao. The fitting results are shown in Table 7. It can be seen that the model of the regional soft environment factor system of small- and medium-sized tourism and hospitality enterprises in Macao has a good fit. The research model is effective.

**TABLE 7:** Fitting index of regional soft environment measurement model (*N* = 317).

Fit	χ^2^	df	χ^2^/df	RMSEA	PGFI	IFI	TLI	CFI
Model	608.67	362	1.681	0.046	0.739	0.934	0.926	0.934
Criteria			<5	<0.08	>0.5	>0.9	>0.9	>0.9

### Confirmatory Factor Analysis of Second-Order Model

**FIGURE 3:**
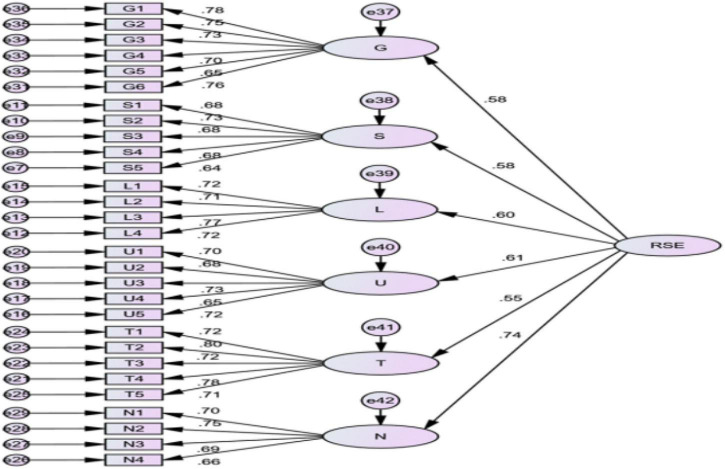
Second-order model of regional soft environment measurement.

Table 8 and Figure 3 show the confirmatory factor analysis results of the second-order factors extracted from the measurement model of the regional soft environment factor system of small- and medium-sized tourism and hospitality enterprises in Macao. It shows that the second-order model of the regional soft environment factor system of small- and medium-sized tourism and hospitality enterprises in Macao is matched well. Therefore, the second-order model can effectively replace the first-order model.

**TABLE 8:** Fitting index of second-order model for regional soft environment (*N* = 317).

Fit	χ^2^	df	χ^2^/df	RMSEA	PGFI	IFI	TLI	CFI
Model	624.803	371	1.684	0.047	0.755	0.932	0.925	0.932
Criteria			<5	<0.08	>0.5	>0.9	>0.9	>0.9

### Regression Analysis of Regional Soft Environment on Competitive Advantage

This article uses the regression analysis method before the structural equation model analysis. The researcher used SPSS version 26.0 statistical software to preliminarily verify the relevant research hypotheses of the role of regional soft environment on the competitive advantage factors of small- and medium-sized tourism and hospitality enterprises in Macao. In regression analysis, the variable is unidimensional, and the average value of the measurement index can replace its value. But the internal consistency coefficient of the measurement index is generally required to be greater than 0.7 (Khalil et al., 2021; Li et al., 2021). In this study, the internal consistency coefficient of the regional soft environment and competitive advantage factor system measurement scale is greater than 0.7. Therefore, it can be replaced by the average value of each measurement item.

This study explores the influence of the regional soft environment on the competitive advantage system of small- and medium-sized tourism and hospitality enterprises in Macao. This study adopts the hierarchical regression method and takes the “Enter” method into the control variables of enterprise type, size, and establishment time. The researcher used the “STEPWISE” method in the various elements of the soft environment. Regression analysis selects the factors that significantly influence the competitive advantage variables and regional soft environment variables of small- and medium-sized tourism and hospitality enterprises in Macao.

### Structural Equation Model Analysis

**FIGURE 4:**
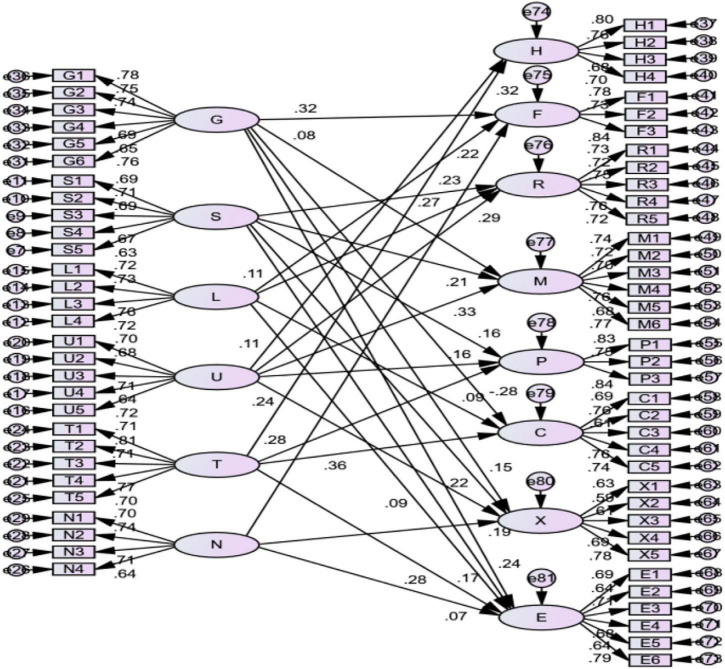
Initial structural equation model.

This study puts forward the theoretical model and related hypotheses of the mechanism of the regional soft environment on the competitive advantage factors of small- and medium-sized tourism and hospitality enterprises in Macao. The researcher drew the corresponding initial structural equation model using AMOS version 24.0 software, as shown in Figure 4.

In the initial model, six exogenous latent variables (i.e., government service environment, social and cultural environment, legal environment, market environment, technology and education environment, and financial environment) are measured by 29 exogenous observable variables. There are eight endogenous latent variables (i.e., human resources, financial resources, relationship resources, management ability, production ability, innovation ability, marketing ability, and entrepreneur ability) measured by 37 endogenous observable variables. The causal relationship between 6 exogenous latent variables and 8 endogenous latent variables is reflected by 14 hypothetical paths.

The influence of the three control variables of enterprise type, size, and establishment time on the dependent variables did not reach a significant level in the regression analysis. Therefore, they are not included in the structural equation model. According to the initial structural equation model diagram, the standardized path coefficient of each measurement model is greater than 0.6, which indicates that the measurement model has a good structure. The normalized path coefficients of all the variables were above 0.5, the corresponding composite reliability (CR) values were greater than the reference critical value of 1.96, and the relevant standardized path was at least at the level of *p* = 0.01. Therefore, it can be considered that the path CR value between most endogenous latent variables and exogenous variables is greater than 1.96. This indicates that the correlation path is statistically significant, at least at the *p* = 0.05 level.

#### Modified Structural Equation Model

**FIGURE 5:**
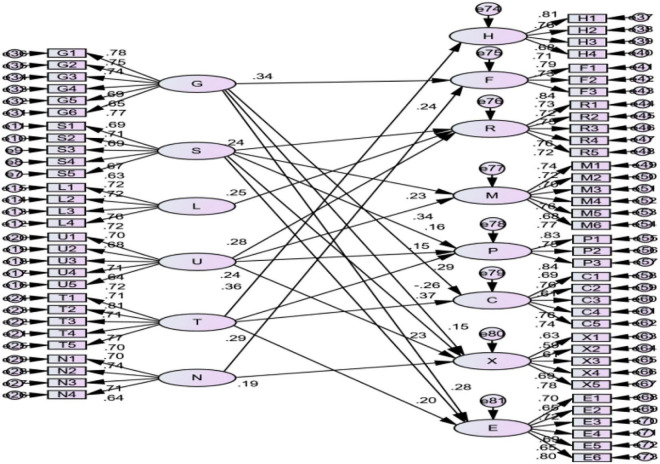
Modified structural equation model.

This study further optimizes the model structure by optimizing the structural equation path, which is insignificant. The optimized model structure is shown in Figure 5.

It can be found that the standardized path coefficient of each measurement model is greater than 0.6, which indicates that the measurement model has a good structure.

Table 9 shows that the value of χ^2^ of the modified model is 3,210.155 (df = 2,059). The value of χ^2^/df is 1.559, which is <2, indicating that the fitting effect is good. Therefore, this study believes that the model fit meets the standard. This shows that data can support the model in this article, and it can be considered that the model structure is very good.

**TABLE 9:** Goodness of fit by modified model.

Fit	χ^2^	df	χ^2^/df	RMSEA	PGFI	IFI	TLI	CFI
Model	3210.155	2059	1.559	0.042	0.710	0.876	0.869	0.875
Criteria			<5	<0.08	>0.5	>0.9	>0.9	>0.9

## Discussion

The tourism and hospitality industry in the world has remained at its boom with the increasing dominance of economy in Macau. The economic dependence in Macau has experienced extreme changes while keeping the service economy at its boom. For understanding the activities of SMEs, this section discusses the study analysis in the light of previous studies. The results revealed that, over the years, economy in Macao had achieved sufficient economies of scale. Indeed, the study analysis showed that booming service sector (i.e., tourism and hospitability) in Macao affects the social, educational, innovation, and technological environment, thus gaining a competitive advantage. As a result, competitive advantage factors deserve considerable attention from researchers and entrepreneurs, causing the tourism boom to increase the economic welfare of Macau.

A total of 317 valid questionnaires and research data were obtained based on the questionnaire survey of small- and medium-sized tourism and hospitality enterprises in Macao. In this study, the exploratory factor analysis and confirmatory factor analysis are used to test the validity and reliability of the regional soft environment and competitive advantage factor system of small- and medium-sized tourism and hospitality enterprises in Macao. The researcher used regression analysis, structural equation model analysis, and one-way ANOVA analysis to verify the theoretical model and research hypothesis of their regional soft environment and competitive advantage factors. Necessary tests and corrections finally determine the theoretical model. The researcher tests the hypothesis through regression and structural equation analyses.

The path hypothesis results of the structural equation model are shown in Table 10. The prior research reveals that government services significantly influence enterprise success. The study shows that the non-market forces have mastery over all kinds of resources. The government ensures the economic development of a country by aggressively contributing toward socio-economic welfare. In particular, the government service environment positively contributes to the survival and development of the enterprise (Amit and Schoemaker, 1993; Shah et al., 2019). The government encourages labor migration by losing travel restrictions, thus helping Macao achieve economic prosperity. Based on this statement, the findings are consistent with the studies (i.e., H1, H2, H3, and H4).

**TABLE 10:** Results of hypothesis.

No.	Hypothesis	Validation results
H1	The government service environment has a significant positive impact on the financial resources of small and medium-sized tourism and hospitality enterprises in Macao.	Supported
H2	The government service environment has a significant positive impact on the management ability of small and medium-sized tourism and hospitality enterprises in Macao.	Supported and Non- significant
H3	The government service environment has a significant positive impact on the marketing ability of small and medium-sized tourism and hospitality enterprises in Macao.	Supported
H4	The government service environment has a significant positive impact on the entrepreneur ability of small and medium-sized tourism and hospitality enterprises in Macao.	Supported
H5	The government service environment has a significant positive impact on the innovation ability of small and medium-sized tourism and hospitality enterprises in Macao.	Not Supported
H6	The social and cultural environment has a significant positive impact on the relationship resources of small and medium-sized tourism and hospitality enterprises in Macao.	Supported
H7	The social and cultural environment has a significant positive impact on the management ability of small and medium-sized tourism and hospitality enterprises in Macao.	Supported
H8	The social and cultural environment has a significant positive impact on the production ability of small and medium-sized tourism and hospitality enterprises in Macao.	Supported
H9	The social and cultural environment has a significant positive impact on the marketing ability of small and medium-sized tourism and hospitality enterprises in Macao.	Supported
H10	The social and cultural environment has a significant positive impact on the entrepreneur ability of small and medium-sized tourism and hospitality enterprises in Macao.	Supported
H11	The legal environment has a significant positive impact on the financial resources of small and medium-sized tourism and hospitality enterprises in Macao.	Supported and Non- significant
H12	The legal environment has a significant positive impact on the relationship resources of small and medium-sized tourism and hospitality enterprises in Macao.	Supported
H13	The legal environment has a significant positive impact on the entrepreneur ability of small and medium-sized tourism and hospitality enterprises in Macao.	Supported and Non- significant
H14	The legal environment has a significant positive impact on the innovation ability of small and medium-sized tourism and hospitality enterprises in Macao.	Non-significant
H15	The market environment has a significant positive impact on the relationship resources of small and medium-sized tourism and hospitality enterprises in Macao.	Supported
H16	The market environment has a significant positive impact on the human resources of small and medium-sized tourism and hospitality enterprises in Macao.	Supported and Non- significant
H17	The market environment has a significant positive impact on the management ability of small and medium-sized tourism and hospitality enterprises in Macao.	Supported
H18	The market environment has a significant positive impact on the production ability of small and medium-sized tourism and hospitality enterprises in Macao.	Supported
H19	The market environment has a significant positive impact on the marketing ability of small and medium-sized tourism and hospitality enterprises in Macao.	Supported
H20	The technology and education environment has a significant positive impact on the human resources of small and medium-sized tourism and hospitality enterprises in Macao.	Supported
H21	The technology and education environment has a significant positive impact on the production ability of small and medium-sized tourism and hospitality enterprises in Macao.	Supported
H22	The technology and education environment has a significant positive impact on the innovation ability of small and medium-sized tourism and hospitality enterprises in Macao.	Supported
H23	The technology and education environment has a significant positive impact on the entrepreneur ability of small and medium-sized tourism and hospitality enterprises in Macao.	Supported
H24	The financial environment has a significant positive impact on the financial resources of small and medium-sized tourism and hospitality enterprises in Macao.	Supported
H25	The financial environment has a significant positive impact on the marketing ability of small and medium-sized tourism and hospitality enterprises in Macao.	Supported
H26	The financial environment has a significant positive impact on the entrepreneur ability of small and medium-sized tourism and hospitality enterprises in Macao.	Supported and Non- significant

Leveraging the competitive advantages in Macao, its unique regional innovative benefit suggests a socioeconomic improvement in sustainability in Macao (Government of Macao, 2016). Accordingly, the results show that H5 does not support the previous literature. The result contradicts the assumption formed in the literature section. Perhaps, in the light of study findings, the current situation of development in Macao requires stability with having numerous strategic opportunities. Apart from the rising crises in the government service support, the government should reinforce the confidence in the economic sector in Macau, thus ensuring sustainable development and competitive advantage. Consistently, the study shows that regional innovation brings numerous benefits for SMEs (Crotti and Misrahi, 2017). The innovation ability plays an integral role in cultivating an innovative cultural environment where the innovation performance largely influences the relationship between the societal members (i.e., individual and enterprise). Perhaps, innovation systems build an interactive association among the economic actors, thereby achieving competitive advantage. Consequently, the government should help the SME clusters, performing regular operations (Jiang and Tan, 2020).

Similarly, analysis shows that H2, H11, H13, H14, H16, and H26 were supported, but not significant. Accordingly, the study shows that the competitive advantage of SMEs largely depends on the innovation ability of knowledge and technical connection between the firms (Guohong and Lijun, 2005). Given this statement, the study results record a positive relationship between the technology and knowledge environment, production ability, innovation ability, and entrepreneur ability. Based on the analysis, H21, H22, and H23 were supported. Perhaps, in addition to these results, other hypotheses are also supported.

## Conclusion

Macao has gained immense economic achievements related to GDP, social welfare, life expectancy, and employment rates. However, in the previous years, the socioeconomic strength in Macau has decreased. The study shows that the rapid shift in economic structure in Macau has entered a period of decline. Consequently, the research topic raises several questions on the broad-based foundation of socioeconomic sustainability and development in Macau. The growing concerns of the research about environmental, social, local, and economic consequences in Macau have made this study a significant contribution to the development in Macau. Accordingly, the findings show that tourism and hospitality industry in Macau has encountered numerous problems regarding the progress in Macau. Indeed, these problems should be addressed, thus gaining long-term sustainable development and competitive advantage. The finding reveals that small- and medium-sized tourism and hospitality enterprises in Macau need proper development. The study witnessed that the support policies issued by the Macao SAR government have not improved the management ability and innovation ability of small- and medium-sized tourism and hospitality enterprises. The support policies and government help to alleviate the market weakness of SMEs.

On the contrary, the government service environment has a significant positive impact on the ability of an entrepreneur. The government has offered free paid courses for business, enhancing the ability of an entrepreneur. The government may give too much support and help to the SMEs. Most of the enterprises are waiting for the support of government. The social and cultural environment in Macao can provide strong potential power for the management ability and business activities.

The law in Macao comes from the Portuguese law system. Therefore, the impact of laws of Macao on the acquisition of financial resources and the promotion of entrepreneur ability and innovation ability is not obvious. The market of Macao is really small, which leads to the market environment having no significant impact on human resources. The local education and technology development cannot help economic development. The market lacks venture investors in Macao. Enterprises cannot obtain funds through angel financing or risk financing.

### Study Suggestions for Small- and Medium-Sized Enterprises

The SMEs in tourism and hospitality in Macao should use its regional soft environment. It can be found that the current regional soft environment of Macao is conducive to the survival and sustainable development of small- and medium-sized tourism and hospitality enterprises in Macao from the research conclusion. Macao has a good social environment, and the Chinese central government also gives special support to economic development in Macau. The Macao SAR government has issued many supportive policies for SMEs. Therefore, the small- and medium-sized tourism and hospitality enterprises in Macao should make good use of this opportunity to develop themselves. They had better try their best to improve the quality of products and services. These enterprises must abandon the erroneous concept of relying on the government for development. A hypothesis shows that the government service environment has a negative impact on the innovation ability of enterprises. This also makes enterprises think about using the support policies of the government. If they do not make good use of the support policies of the government. Every enterprise takes obtaining government funds as the condition for survival and development, and such enterprises will die out slowly in the future.

The small- and medium-sized tourism and hospitality enterprises in Macao should face up to the problems in the development. There are many problems in developing small- and medium-sized tourism and hospitality enterprises in Macao. It can be found that the support policies issued by the Macao SAR government have not improved the management ability and innovation ability of small- and medium-sized tourism and hospitality enterprises from the research results. The market environment does not promote the development of human resources. During this period (COVID-19), the researcher has seen that labor market in Macao is against foreign workers, while enterprises are unwilling to employ local workers in Macao. This fully shows that the development of economy in Macao has not promoted the development of local human resources in Macao. The current financial environment in Macao is developing very well. The financing and lending environment in Macao are relaxed. However, these favorable conditions did not promote Macao entrepreneurs to develop their enterprises actively. Therefore, if the small- and medium-sized tourism and hospitality enterprises in Macao want to survive and develop sustainably, they must face these problems because H16 shows that the market environment in Macao is not significant for the human resource development of enterprises. This problem has a long history. Since 1999, economic development in Macao has depended on foreign capital and the migration of foreign populations. The market and talent scale in Macao are very small. At present, the central government will coordinate the development of Hengqin with Macao. The small- and medium-sized tourism and hospitality enterprises in Macao should grasp this environmental factor. This can be done through the joint development of the two places to realize the further development of the enterprise.

### Suggestion for Macao SAR Government

#### The Government Should Reduce Part of the Cash Support for Small- and Medium-Sized Enterprises

It can be found that the government service environment is not conducive to the development of innovation ability of small- and medium-sized tourism and hospitality enterprises in Macao from the research results. This shows that these enterprises have lost their innovation consciousness and ability because of government support policies. It is consistent with the views of many entrepreneurs during the interview. Therefore, the Macao SAR government should review the support policies for SMEs. The government should expand from the original single financial support and tax relief to more aspects. The government should reduce financial support and turn to technical and sales support. The government can support them through government procurement.

#### The Government and Society Should Be Able to Accommodate New Small- and Medium-Sized Enterprises in Tourism and Hospitality

The government environment and social environment in Macao can significantly affect the resources and capabilities of enterprises. However, from the previous handling of the Uber incident in Macao, the Macao government and society have a low degree of accommodation for emerging tourism and hospitality enterprises. This will affect the long-term development and competitiveness of tourism industry in Macao. Therefore, this article suggests that due to the great impact of the government service environment and social environment, the government level should accommodate new enterprises and new businesses with a more inclusive attitude.

#### The Government Should Promote and Improve the Legal Development

It can be found that the legal environment of Macao is not conducive to the development of financial resources, entrepreneur ability, and innovation ability of small- and medium-sized tourism and hospitality enterprises in Macao from the research results. This shows that the current legal system of Macao lags behind economic development. The small- and medium-sized tourism and hospitality enterprises in Macao need a perfect legal system to survive and sustain development. A few years ago, the exit of the Uber automobile in Macao showed that the laws of Macao do not contain the emergence and development of emerging industries. The laws do not give the new industry a trial-and-error mechanism. Therefore, the law is seriously behind the development of economy in Macao. The government should promote the relevant legal construction. They could learn and refer to the model of Hong Kong and the Mainland of China. They had better timely adjust the legal provisions. The Legislative Council of Macao should also discuss the legislative work instead of developing the legal system in waiting and coping. In the process of mediating effect hypothesis, this article finds that the legal development of Macao is getting better and better. Since Macao is heavily dependent on tourism, the laws of Macao should be formulated and adjusted in line with the small- and medium-sized tourism and hospitality enterprises in Macao.

This way, it could help and promote the survival and sustainable development of small- and medium-sized tourism and hospitality enterprises in Macao. This study conducted extensive field research and verified the validity of the theoretical views and research hypotheses.

## Data Availability Statement

The raw data supporting the conclusions of this article will be made available by the authors, without undue reservation.

## Ethics Statement

Ethical review and approval was not required for the study on human participants in accordance with the local legislation and institutional requirements. The patients/participants provided their written informed consent to participate in this study.

## Author Contributions

All authors listed have made a substantial, direct, and intellectual contribution to the work, and approved it for publication.

## Conflict of Interest

The authors declare that the research was conducted in the absence of any commercial or financial relationships that could be construed as a potential conflict of interest.

## Publisher’s Note

All claims expressed in this article are solely those of the authors and do not necessarily represent those of their affiliated organizations, or those of the publisher, the editors and the reviewers. Any product that may be evaluated in this article, or claim that may be made by its manufacturer, is not guaranteed or endorsed by the publisher.

## References

[B1] Ajaz A. Shenbei Z. Sarfraz M. (2020). Delineating the influence of boardroom gender diversity on corporate social responsibility, financial performance, and reputation. *Logforum* 16 61–74. 10.17270/J.LOG.2020.376

[B2] Alfiero S. Vallone C. (2019). *A Business Model for Sustainable Tourism Experiences: evidence from Albergo Diffuso.* Matera: IFKAD.

[B3] Amit R. Schoemaker P. J. H. (1993). Strategic assets and organizational rent. *Strat. Manage. J.* 14 33–46. 10.1002/smj.4250140105

[B4] Bandura A. Bandura A. (1986). *Social Foundations of Thought and Action: a Social Cognitive Theory.*

[B5] Barney J.. (1991). Firm resources and sustained competitive advantage. *J. Manag. Stud.* 17, 99–120

[B6] Castellani V. Sala S. Mirabella N. (2015). Beyond the throwaway society: a life cycle-based assessment of the environmental benefit of reuse. *Integr. Environ. Assess. Manage.* 11 373–382. 10.1002/ieam.1614 25557152

[B7] Chance D. M. (2019). *Organizational Structure and Corporate Governance of Financial Risk Management.* Singapore: World Scientific.

[B8] Cope J. Kempster S. Parry K. (2011). Exploring Distributed Leadership in the Small Business Context. *Int. J. Manag. Rev.* 13 270–285. 10.1111/j.1468-2370.2011.00307.x

[B9] Crotti R. Misrahi T. (2017). *The Travel and Tourism Competitiveness Report 2017. Paving the Way for a More Sustainable and Inclusive Future.* Geneva: World Economic Forum.

[B10] Gersick C. J. G. (1994). Pacing Strategic Change: the Case Of A New Venture. *Acad. Manag. J.* 37 9–45. 10.2307/256768

[B11] Government of Macao (2016). *The Five-Year Development Plan of the Macao Special Administrative Region (2016-2020).* Portugal: Government of Macau. https://policy.asiapacificenergy.org/sites/default/files/plano_quinquenal_en.pdf

[B12] Grant R. M. (1996). Toward a knowledge-based theory of the firm. *Strat. Manag. J.* 17 109–122. 10.1002/smj.4250171110

[B13] Guohong Z. Lijun L. (2005). Research on the influence of industry university research on Enterprise Competitiveness – Based on the questionnaire survey and analysis of 1639 small and medium-sized enterprises. *Res. Dev. Manag.* 7, 64–68

[B14] Guoquan C. (2001). The relationship between organization and environment and organizational learning. *J. Manag. Sci.* 5, 49–61

[B15] Hardwick P. Dou W. (1998). The Competitiveness of EU Insurance Industries. *Serv. Industr. J.* 18 39–53. 10.1080/02642069800000003

[B16] Jiang Q. Tan Q. (2020). Can government environmental auditing improve static and dynamic ecological efficiency in China? *Environ. Sci. Poll. Res.* 27 21733–21746. 10.1007/s11356-020-08578-7 32279266

[B17] Jing-lian W. (2011). Accelerating transformation in the pattern of growth is the only way for china to get out of the international financial crisis. *New Economy* 000, 34–37.

[B18] Khalil M. Khawaja K. F. Sarfraz M. (2021). The adoption of blockchain technology in the financial sector during the era of fourth industrial revolution: a moderated mediated model. *Qual. Quant.* 2021 1–18. 10.1007/s11135-021-01229-0

[B19] Kokkali P. Koutsouris A. (2009). Cognitive components of rural tourism destination images: the case of Lake Plastiras, Greece. *Tour. Int. Multidiscipl. J. Tour.* 4 1–5.

[B20] Larsson J. P. Thulin P. (2019). Independent by necessity? The life satisfaction of necessity and opportunity entrepreneurs in 70 countries. *Small Bus. Econ.* 53 921–934. 10.1007/s11187-018-0110-9

[B21] Li N. Bao S. Naseem S. Sarfraz M. Mohsin M. (2021). Extending the Association Between Leader-Member Exchange Differentiation and Safety Performance: a Moderated Mediation Model. *Psychol. Res. Behav. Manag.* 14:1603. 10.2147/PRBM.S335199 34675701 PMC8504862

[B22] Loasby B. J. (1995). Running a Business: an Appraisal of Economics, Organization and Management by Paul Milgrom and John Roberts. *Industr. Corpor. Change* 4 471–489. 10.1093/icc/4.2.471 30476010

[B23] Lorenzo J. R. F. Rubio M. T. M. Garcés S. A. (2018). The competitive advantage in business, capabilities and strategy. What general performance factors are found in the Spanish wine industry? *Wine Econ. Pol.* 7 94–108. 10.1016/j.wep.2018.04.001

[B24] Lubit R. (2010). Tacit Knowledge and Knowledge Management: the Keys to Sustainable Competitive Advantage. *Organiz. Dyn.* 29 164–178.

[B25] Macao Yearbook of Statistics (2019). Available online at: https://www.dsec.gov.mo/en-US/Home/Publication/YearbookOfStatistics (accessed May 11, 2021).

[B26] Nachum L. Jones G. G. Dunning J. H. (2001). The international competitiveness of the UK and its multinational enterprises. *Struct. Change Econ. Dyn.* 12 277–294. 10.1057/s41267-020-00347-5 32836502 PMC7330527

[B27] Ostroff F. (1999). *The Horizontal Organization: What the Organization of the Future Actually Looks Like and How it Delivers Value to Customers.* New York: Oxford University Press. ISBN: 9780195121384

[B28] Porter M. E. (1997). Competitive Strategy. *Measur. Bus. Excell.* 1 12–17. 10.1108/eb025476

[B29] Rajan A. (2002). *Building Tomorrow’s Company: a Guide to Sustainable Business Success.* United States: Kogan Page.

[B30] Ruosen Y. Sha X. (2019). Political connection, institutional environment and innovation performance of Family Enterprises – an explanation from the perspective of social emotional wealth theory. *Sci. Technol. Progr. Countermeasur.* 6 36–46.

[B31] Sarfraz M. Shah S. G. M. Ivascu L. Qureshi M. A. A. (2021). *Explicating the Impact of Hierarchical CEO Succession on Small-medium Enterprises’ Performance and Cash Holdings.* United States: John Wiley & Sons, Ltd. 10.1002/ijfe.2289

[B32] Schroeder R. G. Bates K. A. Junttila M. A. (2002). A resource-based view of manufacturing strategy and the relationship to manufacturing performance. *Strat. Manag. J.* 23 105–117. 10.1002/smj.213

[B33] Shah S. G. M. Sarfraz M. Fareed Z. Rehman M. A. Ur Maqbool A. Qureshi M. A. A. (2019). Whether CEO Succession Via Hierarchical Jumps is Detrimental or Blessing in Disguise? Evidence from Chinese Listed Firms. *Zagreb Int. Rev. Econ. Bus.* 22 23–41. 10.2478/zireb-2019-0018

[B34] Shah S. G. M. Sarfraz M. Ivascu L. (2021). Assessing the interrelationship corporate environmental responsibility, innovative strategies, cognitive and hierarchical CEO: a stakeholder theory perspective. *Corpor. Soc. Responsibil. Environ. Manag.* 28 457–473.

[B35] Singh J. V. (1994). Organizational Niches and the Dynamics of Organizational Mortality. *Am. J. Sociol.* 100 346–380. 10.1086/230540

[B36] Zhang P. Wang N. Yang L. Zhang X. Liu Q. (2020). Evaluation and sensitivity analysis of the ecosystem service functions of haze absorption by green space based on its quality in China. *Nat. Conserv.* 40 93–141. 10.3897/natureconservation.40.23017

[B37] Zhang Q. Z. (2019). The construction of core competence in accelerating the development of new economy. *Res. Finan. Econ. Issues* 28, 17–23.

